# Assessing the Validity of Sexual Behaviour Reports in a Whole Population Survey in Rural Malawi

**DOI:** 10.1371/journal.pone.0022840

**Published:** 2011-07-27

**Authors:** Judith R. Glynn, Ndoliwe Kayuni, Emmanuel Banda, Fiona Parrott, Sian Floyd, Monica Francis-Chizororo, Misheck Nkhata, Clare Tanton, Joanne Hemmings, Anna Molesworth, Amelia C. Crampin, Neil French

**Affiliations:** 1 Faculty of Epidemiology and Population Health, London School of Hygiene & Tropical Medicine, London, United Kingdom; 2 Karonga Prevention Study, Chilumba, Malawi; 3 Centre for Sexual Health and HIV Research, Research Department of Infection and Population Health, University College London (UCL), London, United Kingdom; University of Cape Town, South Africa

## Abstract

**Background:**

Sexual behaviour surveys are widely used, but under-reporting of particular risk behaviours is common, especially by women. Surveys in whole populations provide an unusual opportunity to understand the extent and nature of such under-reporting.

**Methods:**

All consenting individuals aged between 15 and 59 within a demographic surveillance site in northern Malawi were interviewed about their sexual behaviour. Validity of responses was assessed by analysis of probing questions; by comparison of results with in-depth interviews and with Herpes simplex type-2 (HSV-2) seropositivity; by comparing reports to same sex and opposite sex interviewers; and by quantifying the partnerships within the local community reported by men and by women, adjusted for response rates.

**Results:**

6,796 women and 5,253 men (83% and 72% of those eligible) consented and took part in sexual behaviour interviews. Probing questions and HSV-2 antibody tests in those who denied sexual activity identified under-reporting for both men and women. Reports varied little by sex or age of the interviewer. The number of marital partnerships reported was comparable for men and women, but men reported about 4 times as many non-marital partnerships. The discrepancy in reporting of non-marital partnerships was most marked for married women (men reported about 7 times as many non-marital partnerships with married women as were reported by married women themselves), but was only apparent in younger married women.

**Conclusions:**

We have shown that the under-reporting of non-marital partnerships by women was strongly age-dependent. The extent of under-reporting of sexual activity by young men was surprisingly high. The results emphasise the importance of triangulation, including biomarkers, and the advantages of considering a whole population.

## Introduction

Monitoring sexual behaviour is a key component of second generation surveillance for HIV, and an important tool in understanding HIV risk behaviours. But it is fraught with difficulties [1], [2]. Under-reporting of high risk or socially disapproved behaviour is widespread, especially by women [3], [4].

Studies should reduce reporting bias as far as possible, for example by careful wording of questions, or using different interviewing techniques as an alternative to the traditional face-to-face interviews [5], [6]. But bias will always remain, and it is important to estimate the extent of the bias by triangulating information from different sources [3], [7].

In a sexual behaviour survey in northern Malawi within a demographic surveillance site, we attempted to increase reporting by using a series of probing questions if the respondent initially denied any sexual activity [8]. We triangulated the results using biological markers and by comparison with in-depth interviews [2]. To measure the extent and pattern of under-reporting of non-marital partnerships by women, the reports of men and women in the population were compared using information on age and marital status of partners given by respondents [9].

## Methods

The study was conducted within the demographic surveillance system of the Karonga Prevention Study, which was started in 2002 and covers a population of about 33000 individuals with continuous registration of births, deaths and migrations, and annual updates [10]. After extensive piloting, a house-to-house sexual behaviour survey was started in 2008. This lasted 12 months and included all adults aged 15–59 following written consent. Each interview was conducted by one interviewer with one respondent in private in the local language, with information recorded on to paper forms. Details of sexual behaviour included partner details for all non-marital partners in the last 12 months, and current and past spouses. Separate written consent was sought for HIV and Herpes simplex type-2 antibody (HSV-2) testing.

Ethics statement: Ethics approval for the study was received from the Health Sciences Research Committee, Malawi, and the ethics committee of the London School of Hygiene & Tropical Medicine, UK. Before the start of the demographic surveillance the Traditional Authority that covers the area, and all village headmen and traditional advisors in the study area were informed about the aims of the study and the nature of the data to be collected, and their approval and verbal consent was sought. All household members were given a similar explanation and interviews were only conducted if verbal consent was given by the household head and by the respective household members. The consent for the demographic surveillance was recorded by the interview sheet being filled. Refusals were recorded in field registers. During the baseline census 15 households did not provide verbal consent and have consequently been excluded from the study. The socio-demographic data for this study come from the basic demographic surveillance for which the ethics committees agreed that written consent was not needed. For the sexual behaviour survey and for HIV and HSV-2 testing, separate individual written consent was sought.

Information on spouses was collected in two separate ways. As part of the demographic surveillance individuals were asked about their current spouse (or spouses: this is a polygynous society), and these spouses, most of whom were co-resident, were identified. The demographic surveillance questionnaire could be completed by an informant, usually a close relative, if the individual was not present. As part of the sexual behaviour questionnaire, conducted by a separate field team an average of 6 weeks later, individuals were asked in more detail about their current spouse(s) and any marriages that had ended in the last 12 months. Marriage was defined as “married or living as married”.

In an attempt to improve reporting of sexual activity the initial question on “ever having had sexual intercourse” explicitly included one-off and unwanted sex. Those who said “no” were asked a series of four follow-up questions: sex with a boyfriend/girlfriend or someone they expected to marry; sex with a friend or acquaintance; sex with someone they had just met; unwanted sex. The HIV and HSV-2 status was assessed in those who reported never having had sex. HSV-2 has previously been shown to be good marker of sexual activity [11]. HSV-2 tests were only done on those under 30 years as prevalence reaches a plateau at older ages [12]. We used a type-2 specific enzyme immunoassay (Kalon Biological Ltd, Surrey, UK), which has the highest sensitivity and specificity of commercially available assays on African samples [13].

To assess the extent of underreporting of non-marital partners by women, the number and characteristics of non-marital partners reported by the men was compared with the number and characteristics of women reporting non-marital partners. For the men, details of all partners reported in the last 12 months were used to derive the numbers of partners overall, the number living within the demographic surveillance area, and the number by age group and marital status. Age of the partner was reported either as an exact age, or as an estimated age difference from the respondent's age. For the women, the number of partners in the last 12 months reported in total and living within the demographic surveillance area was recorded overall and by age group and marital status of the woman interviewed. For both men and women these reported numbers were adjusted according to the sex-specific participation rates in the study, to derive estimates of the total number of reported partners in the area.

Since one reason for under-reporting of non-marital partners by women may be that they are more likely than men to describe a partnership as a marriage [9], numbers of marital partners were also compared, and the proportion of identified husbands who considered themselves married was assessed.

The reporting of key variables was assessed by sex and age of the interviewer, to see if this influenced the responses given. The interviewers have been trained in HIV counselling as well as interviewing and some have considerable research experience. Some are trained medical assistants.

An in-depth qualitative study of sexual behaviour and fertility at an antiretroviral (ART) clinic, taking place between June and August 2009, provided data which could be compared with the responses the same individuals had given in the sexual behaviour questionnaire. These HIV positive individuals were selected from among those attending the clinic, stratified by clinical stage at presentation and sex. The numbers and classification of partners in the lifetime and in the last 12 months mentioned during these interviews were compared with the questionnaire responses from the sexual behaviour questionnaire in 2008–9. The interviews, which often involved meeting at the clinic and subsequently the household, incorporated high levels of probing, particularly about current relationships.

## Results

Of 8232 women and 7338 men aged 15–59 resident in the surveillance area, 7245 women and 5725 men were found and seen; 6825 women and 5283 men agreed to be interviewed about their sexual behaviour, and 6796 women and 5253 men were interviewed. The response rate (compared to those eligible) for women was thus 83% and for men 72%, and varied little by age. All but 6 women and 3 men interviewed responded to the question on ever having had sex. A total of 88.7% (6024) of the women and 75.5% (3966) of the men reported that they had ever had sex. (Median age at first sex is higher for men than for women in this population [14].).

### Ever had sex

**Figure 1:**
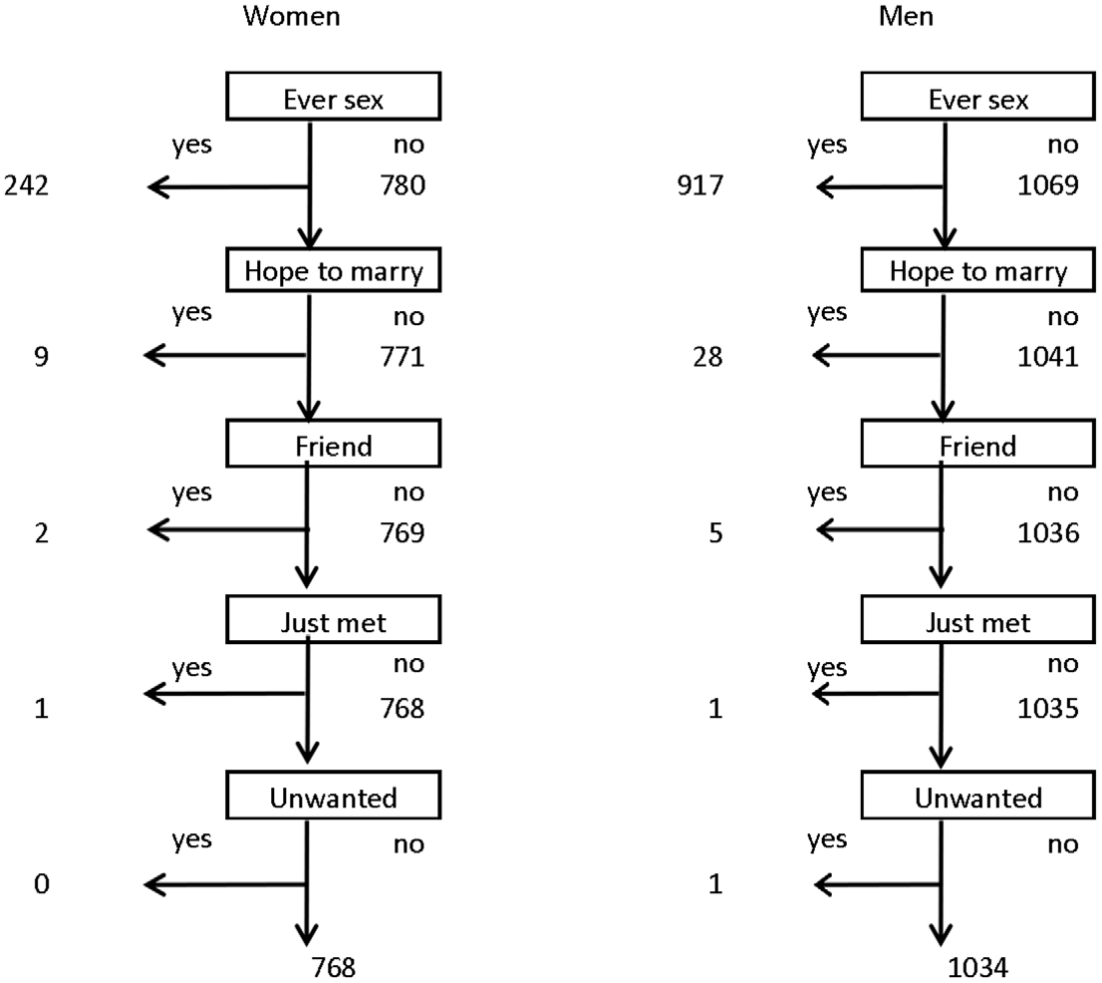
Additional reporting of ever having had sex among unmarried individuals following probing questions. The questions that were asked (in the local language) were the following. 1. Have you ever had sexual intercourse? Think carefully. Maybe it was a man/woman you had sex with only once, maybe it was with someone who was just a friend, with someone you had just met. Even someone who may have forced you to have sex, or someone who gave you gifts/money in order to have sex. 2. You haven't even had sex with a spouse or a boyfriend, or someone you expected to marry? 3. How about with someone who was just a friend or an acquaintance? 4. How about with someone you had just met? 5. How about with someone who forced you to have sex with him/her?

Among 1022 women who had never married, 242 (24%) reported “yes” to the first question about sexual activity, another 9 reported “yes” only after the second question, with a further 3 individuals reporting “yes” after subsequent questions (figure 1). Similarly for the 2040 never married men, 917 (45%) said “yes” initially, the second question picked up a further 28 and the other questions picked up 7 more individuals. Thus for women 1.5% (12/780), and for men 3.3% (35/1069), of those initially denying any sexual activity, changed their answer after additional questions.

Among those who reported never having had sex, 429 women had HSV-2 results of whom 31 (7.2%) were HSV-2 positive. Four additional women had positive HIV results. For the men, 597 who reported never having had sex had HSV-2 results, of whom 52 (8.7%) were positive. Two additional men had positive HIV results.

### Non-marital partners

**Figure 2:**
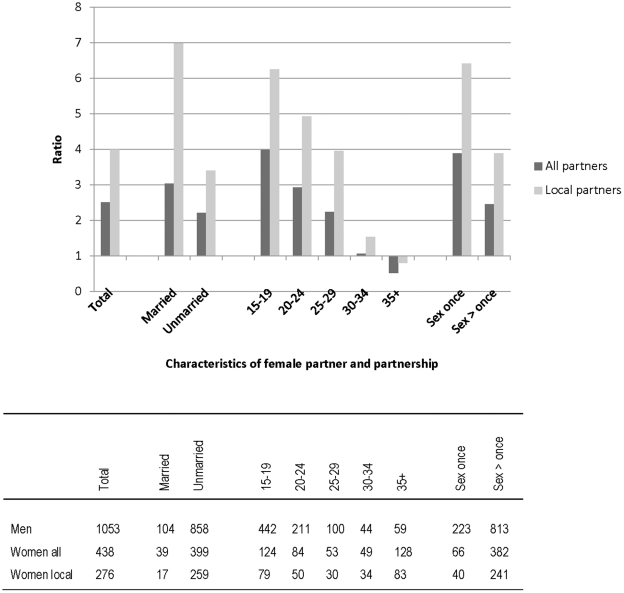
Ratio of local non-marital partners reported by men compared to local or all non-marital partners reported by women, by characteristics of the women and partnership. The table shows the actual number of partners reported. The ratios were adjusted for participation rates of men and women, assuming constant proportions in each group (eg married and unmarried).

In the sexual behaviour survey the men reported details on a total of 1440 non-marital partners in the previous 12 months (table 1). Exact ages were reported for 887, estimates for 480, and place of residence for 1295: 1053 lived within the demographic surveillance area. Of those female partners living in the area, 104 were married and 858 unmarried. 96 of the local partners were reported to be aged under 15 years. The age distribution of the remaining local partners is shown in figure 2. The women interviewed reported a total of 438 non-marital partners in the last 12 months, of whom 276 lived within the surveillance area (residence unknown for 88). Overall 39 of the partnerships were reported by married women (17 of those within the local area).

**Table 1:** Number of partnerships reported in the sexual behaviour questionnaire.

	Men	Women
Current marriages	3602	4754
Marriages ended in last 12 months	123	164
Non-marital partners in last 12 months	1440	438
Total	5165	5356
Current marriages		
with spouses age 15–59	3504	4232
with spouses age 15–59 in local area	3456	4038
+adjusted for response rate	4800	4865

To compare the reports from men and women, only the local partners of the men were considered, and the reported female partners under 15 years were excluded, as women of this age were not interviewed. The ratio of the reported partners was calculated after adjusting for the response rates for men and women. The results are shown in figure 2, using both the total partner numbers reported by the women, and those known to live within the surveillance area. Overall, men reported 2.5 times as many non-marital partners as did the women. The ratio was slightly higher for married women (3.0) than unmarried women (2.2). The ratio was much higher for the younger women (4.0), and fell below 1 in the oldest age group. The ratio was higher for one-off sexual unions (3.9) than for longer partnerships (2.5). Restricting the women's partners to those known to live locally increased the ratio by about 50% in most groups, and more for married women (figure 2).

Men reported that 15 of the partners in the surveillance area were bargirls and 22 were strangers. Only one woman reported sex with a stranger, outside the surveillance area, and no woman reported sex with more than four non marital partners in the previous 12 months.

### Marital partners

The total number of marital partners reported by men and women in the sexual behaviour survey is shown in table 1, together with the numbers known to be within the age group 15–59 and living in the demographic surveillance area. After adjusting for response rates the numbers reported by men and women were similar (4800 reported by men, 4865 by women).

Of the women who reported that they were married in the sexual behaviour survey 3629 were also interviewed in person during the demographic surveillance. 3540 (97.6%) reported that they were married, 57 that they were divorced or separated, 19 that they were never married and 10 that they were widowed (not recorded for 3). All but 6 of the 3540 women gave details of a spouse that allowed them to be identified. Of these identified spouses, 2444 were men who were interviewed in the sexual behaviour questionnaire, and 2411 (98.7%) said they were currently married. 984 of these married men were also interviewed in person in the demographic surveillance interview, and for 971 (98.7%) the spouses identified by husband and wife matched. If we assume that the proportions confirmed are similar for all the 4754 women who said they were currently married (table 1), we can estimate that 62 of the husbands (1.3%) would not say they were married at all, and a further 61 (1.3% of 4754–62) would not recognise a woman who claimed to be married to them as their spouse.

### Characteristics of interviewer

The sex and age of the interviewer had only a minor influence on reporting (table 2). Although some of the differences reached statistical significance because of the large numbers, the magnitude of the differences was small. In general slightly more sexual partners were reported to older interviewers by both men and women. This was not explained by older age of the participants, and after adjusting for age of the respondent, age of the interviewer rather than age difference from the respondent was associated with increased reporting (not shown).

**Table 2:** Influence of interviewer characteristics on reporting.

	Interviewer characteristics					
	Same sex	Opposite sex	*p*	Age<30	Age≥30	*p*
**Men**						
n	3734	1519		1988	3265	
% ever sex	80.3	80.3	*1.0*	78.9	81.2	*0.05*
Median number lifetime partners	3	3		3	3	
% with ≥5 lifetime partners (if sexually active)	31.7	32.6	*0.6*	29.4	33.4	*0.008*
% married men with ≥1 non-marital partner last 12 months	10.8	11.8	*0.5*	10.0	11.7	*0.2*
**Women**						
n	2475	4321		2434	4362	
% ever sex	89.9	88.1	*0.03*	88.3	88.9	*0.4*
Median number lifetime partners	2	2		2	2	
% with ≥3 lifetime partners (if sexually active)	20.5	19.4	*0.3*	18.6	20.5	*0.08*
% married women with ≥1 non-marital partner last 12 months	0.6	0.7	*0.6*	0.6	0.7	*0.7*

### Comparison with in-depth interviews among ART clinic attendees

29 women and 20 men participated in both the sexual behaviour study and the in-depth interviews, median age 36 for both. Only 9 of the women and 12 of the men were currently married. Two participants reported they had never had sex in both studies: a 15 year old male described episodes of ill health since he was 6 years old; a 26 year old female lived with her mother and maintained she had never had a boyfriend or husband, or given birth to a child.

Most individuals identified the same number of partners in the in-depth interviews as in the survey (table 3). Currently divorced, widowed and never married women freely reported in the in-depth interviews the details of their relationships with boyfriends, who were often married men. Four unmarried women and one married woman reported more lifetime partners in the in-depth interviews, the married woman reporting a series of extra-marital affairs in past years when questioned about whether she thought her husband was the source of HIV infection. One man reported fewer total partners in the survey than in the in-depth interviews, and two men reported a higher number of life-time partners in the survey than in the in-depth interviews (table 3).

**Table 3:** Comparison of responses in in-depth interviews vs sexual behaviour survey for individuals who were interviewed in both.

	Women (n = 29)	Men (n = 20)
**Lifetime partners**		
Report more partners	5	1
Report fewer partners	0	2
Same	23	16
Missing	1	1
**Last 12 month non-marital partner**		
Report more partners	3	1
Report fewer partners	0	1
Same	26	17
Missing	0	1

For partners in the last 12 months, three unmarried women described having non-marital partners in interview which were not accounted for in the survey or by differences in interview dates. (A further 2 women may have been describing new partners subsequent to marital change as they reported being married at the time of the survey.) One married man reported extra-marital affairs in the last 12 months in the survey but described this as being “impossible” in interview.

## Discussion

In common with all other surveys of sexual behaviour we found evidence of under-reporting of sexual activity by women: sexually transmitted infections in those who denied sexual activity, reporting of additional activity in in-depth interviews, and reporting of non-marital partnerships that did not correlate with those reported by men.

Some of the discrepancy between reports from men and women could be due to exaggeration by men [9], and we found some suggestion of that in the in-depth interviews. However we also found evidence of under-reporting of partnerships for men as well as women, as has been found elsewhere [2]. The probing questions in unmarried individuals who initially denied sexual activity picked up a higher proportion of extra reports for the men (3.3%) than for the women (1.5%). HSV-2 testing also showed a higher proportion of HSV-2 positive “virgins” among men than women (8.7% vs 7.2%) which is surprising as HSV-2 prevalence is higher in women than in men in this and other populations [12]. False positives are possible, although the test has high specificity (98%) [13]. HIV testing suggested further under-reporting, although it is possible that some of these cases were due to vertical transmission (both men and two of the women were 15 years old).

Since we had data on all reported partners in the last 12 months in a whole population we had the unusual opportunity of being able to triangulate the partner reports of men and women directly. Considering partners who lived locally, who should have been fully included, men reported 4 times as many non-marital partners as did the women (figure 2). The biggest shortfall in reporting was seen for married women, with men reporting 7 times as many partnerships with married women, as were reported by the women themselves. This is in marked contrast to rural Tanzania, where few men reported partnerships with married women [9].

There was a striking trend in the ratio of men's and women's reports by age (figure 2), with high ratios where the female partner was young, but a ratio below one in the oldest age group. The trend with age has been noted in other settings, but in cluster rather than whole population surveys where the comparison is less direct [3]. The trend could suggest a greater reluctance to report non-marital partnerships in younger women, and/or a tendency for men to under-estimate the age of their partner. There could also be some over-reporting by older women, but the partners for whom the men did not know the ages were not included in this calculation, which will underestimate the ratio.

There was more discrepancy in male and female reports of one-off sexual encounters than of longer term partnerships. Although bargirls and strangers made up only a small proportion of reported partners for the men, the absence of any woman reporting more than four partners suggests failure to include sex workers in the study, or for them to report accurately. The demographic surveillance is limited to people who are members of household and excludes short-term visitors.

The in-depth interview results, which were mainly from older individuals, confirmed the survey results for most participants, but did find evidence of both under reporting (for women and men) and over reporting (for men). This is a selected group who were on ART, so may be less reluctant to talk about partners than are individuals in the general population. In these interviews it was noted that women were more reluctant to discuss local partners than partners from outside the area.

In-depth interviews are also liable to under-reporting. Since husbands are expected to divorce adulterous wives [15] the risk for a married woman in describing extra-marital partners may be too high, especially if she is young and has had few children to bring security within the marriage. One older male participant had recently sent his third wife back to her parents for this reason. Married men's extra-marital relationships are more or less sanctioned by the institution of polygyny. Younger men and women's relationships may come under greater scrutiny and control by their families. Four men reported in the in-depth interviews having been forced to marry school-girl brides.

Marriage in sub-Saharan Africa has been described as ‘a process, not an event’ [16]. The majority of marriages in this population are informal (unpublished results), ie without ceremony, involvement of elders or payment of the traditional bride price. Different classifications of a relationship as a marriage by the men and women involved would therefore be possible. In the in-depth interviews some women described men with whom they had longer term relationships as ‘like a husband’ but also noted that they did not live in the man's household but in their own or parental compounds. These relationships were not described as marriages in the survey. However the differences in reporting in non-marital partnerships by men and women did not seem to be explained by different tendencies to classify relationships as marriage. After excluding marital partners outside the age ranges and living outside the area, the numbers reported by men and women were similar. And where information was available on the actual identities of spouses there was good corroboration of the reports. The percentage of marriages reported by women but not confirmed was low. We can estimate that overall this might have led to an extra 123 partnerships being classified as non-marital. This would increase the total number of non-marital partners reported by women by 28%; not enough to make much difference to the discrepancies with the reports by the men.

In making estimates of partner numbers in the population we allowed for non-participation, but this assumes that those not interviewed would have similar patterns to those included, which is unlikely to be the case. Participation rates were quite high, but it is likely that some high risk individuals with multiple partners (including commercial sex workers) were not included as they chose to avoid our interviewers or were temporary residents in the district, which would explain some of the discrepancies. By including a whole population, and considering partners known to live within it, we have avoided the usual problem in partner number comparisons of partners outside the survey area.

It is usually recommended that interviewers should be of the same sex and similar age as the interviewee [17]. We were unable to do this due to the difficulty in recruiting sufficient female interviewers, and logistic constraints. The small effect of sex or age of the interviewer on the results was surprising, and suggests that sex and age matching is not a priority in this setting.

This study emphasises the importance for caution in interpreting sexual behaviour data, the need for triangulation, including biological data if possible, and the problem of under-inclusion of high risk women in general surveys of this sort.
